# T2 Mapping of the myocardium, a quantitative tool for assessment of myocarditis

**DOI:** 10.1186/1532-429X-14-S1-P177

**Published:** 2012-02-01

**Authors:** Darshit Thakrar, Jeremy Collins, Rahul Rustogi, Aya Kino, Sven Zuehlsdorff, James Carr

**Affiliations:** 1 Cardiovascular imaging, Northwestern Memorial hospital, Chicago, IL, USA; 2 Siemens Healthcare, Chicago, IL, USA

## Background

To quantify myocardial T2 value in patients with myocarditis and correlate the distribution of abnormal T2 values with the extent of macroscopic late gadolinium enhancement (LGE).

## Methods

25 patients with myocarditis were retrospectively evaluated for the utility of T2 mapping in diagnosing myocarditis. Patients with elevated troponins, negative coronary angiogram, and atypical LGE were diagnosed as acute myocarditis. Patients with normal troponins and macroscopic LGE at the time of cardiac MRI were diagnosed as remote myocarditis. As per our institutional protocol, T2 mapping sequences were performed in all cases with suspected myocarditis in addition to standard LGE images on 1.5 T scanner (Magnetom Aera and Avanto, Siemens medical solutions). T2 mapping was performed on three short axis images (base, mid chamber, and apex), yielding 16 myocardial segments for analysis (AHA segments). Single 4 chamber view image was obtained in addition. Minimum, peak and mean segmental T2 values were calculated by the first reader. Average segmental T2 values were documented along with documentation of the number of segments with elevated T2 values. The presence or absence of LGE was documented by a second reader blinded to the T2 results. Average segmental T2 values were then correlated with troponin levels at the time of the MRI examination.

## Results

In patients with acute myocarditis, mean T2 values were elevated in segments showing LGE (average T2 value of 70 msec). The T2 values were also elevated in myocardial segments with no macroscopic LGE (avg 60 msec). On an average, there were 6 additional segments that showed elevated T2 values and no macroscopic LGE. In patients with remote myocarditis, the T2 values were normal in areas of LGE.

## Conclusions

T2 mapping can be utilized as an objective tool for diagnosing acute myocarditis. In addition, T2 values were normal in patients with normal cardiac enzymes and a typical pattern of myocarditis associated LGE. Given the non contrast nature of this technique, normal T2 values may exclude a diagnosis of acute myocarditis in patients with renal insufficiency.

## Funding

None.

**Figure 1:**
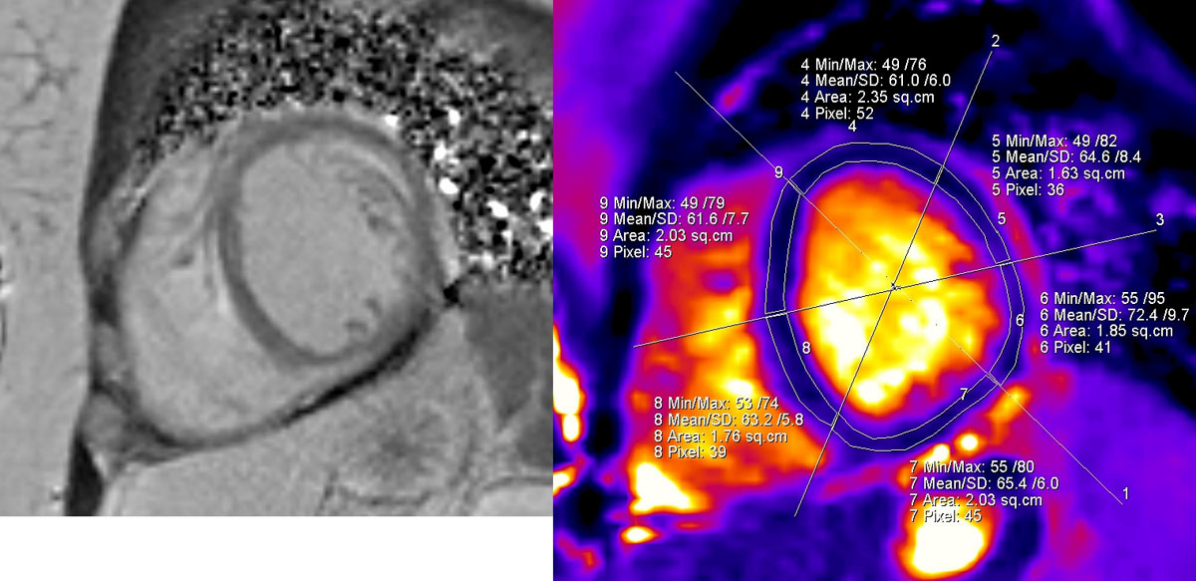
SAX mid chamber views. IR LGE image on the left showing subendocardial LGE in the anterolateral wall and subepicardial LGE in the inferior septum. Corresponding T2 map on the right shows elevated mean T2 values (> 60) in all 6 segments at that level.

**Figure 2:**
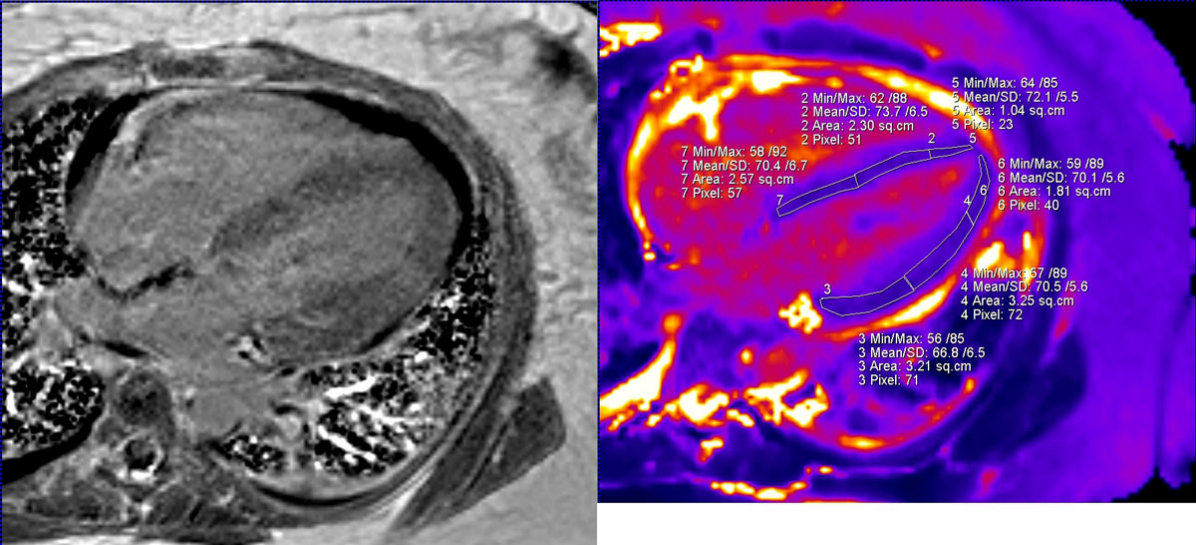
4 Chamber views. IR LGE image (left) showing a small patchy area of LGE with no other area of obvious LGE. T2 map (right) shows elevated T2 values in all the visualized myocardial segments. This patients troponin on the day of MRI was 1.26 with the EF of 39%.

